# Explorative study on patient’s perceived knowledge level, expectations, preferences and fear of side effects for treatment for allergic rhinitis

**DOI:** 10.1186/2045-7022-2-9

**Published:** 2012-05-29

**Authors:** Peter W Hellings, Fabienne Dobbels, Kris Denhaerynck, Mark Piessens, Jan L Ceuppens, Sabina De Geest

**Affiliations:** 1 Laboratory of Experimental Immunology, University Hospitals Leuven, Kapucijnevoer 33, 3000, Leuven, Belgium; 2 Department of Otorhinolaryngology-Head and Neck Surgery, University Hospitals Leuven, Kapucijnevoer 33, 3000, Leuven, Belgium; 3 Department of Internal Medicine, Allergy Division, University Hospitals Leuven, Leuven, Belgium; 4 Center for Health Services and Nursing Research, Katholieke Universiteit Leuven, Leuven, Belgium; 5 Institute of Nursing Science, Faculty of Medicine, University of Basel, Basel, Switzerland

**Keywords:** Allergy, Rhinitis, Treatment, Patient reported outcomes, Preferences, Side-effects

## Abstract

**Background:**

In spite of the high prevalence of allergic rhinitis (AR) and the evidence-based guidelines for treatment, little is known about the patients’ perceived knowledge level, expectations, preferences for treatment, and fear for side effects of treatment for AR. This study aimed at gaining insight into these patient-related factors.

**Methods:**

This explorative cross-sectional survey study included a convenience sample of 170 patients with rhinitis and clinical suspicion of allergy at the department of Otorhinolaryngology and Allergology. Patients’ perceived knowledge level, expectations, patient preferences, and fear of side effects of allergy treatment were collected via a self-report questionnaire developed for the purpose of this study.

**Results:**

22% of all patients (38/170) reported to have knowledge about anti-allergic treatment. 40% (55/170) of rhinitis patients expected to be cured by the prescribed treatment, whereas 43% (73/170) of patients expected suppression of allergic symptoms. Nasal spray was the preferred route of anti-allergic drug administration in 30% (52/170) of patients, followed by oral treatment (24%; 42/170), combination therapy (16%; 30/170), and injection therapy (15%; 27/170). More patients would choose a combination treatment with step-down approach (31%; 53/170) than mono-therapy with a step-up approach (20%; 34/170). Fear for side effects was reported mainly for nasal corticosteroids (48%; 81/170) and less for oral antihistamines (33%; 36/170), leucotriene antagonists (21%, 36/170) and immunotherapy (19%, 33/170).

**Conclusions:**

Patients consulting for rhinitis have high expectations of anti-allergic treatment, prefer a nasal spray above oral treatment, prefer combined treatment rather than monotherapy, and fear adverse events of anti-allergic treatment.

## Background

Allergic rhinitis (AR) represents a common airway disease, with an estimated prevalence of 30% of the total population in Europe and the US suffering from allergen-induced nasal obstruction, rhinorrhoea, sneezing, itchy nose and/or itchy eyes ([1]) ([2]). Thanks to the expansion of studies on treatment of allergic disease during the last decades, evidence-based guidelines for treatment of AR are nowadays available([1]). The ARIA document provides an extensive overview on clinical effectiveness of available treatment options. It is however, recommended that clinicians tailor their treatment to the individual patient. More specifically, disease-related aspects of AR like severity of symptoms and presence of ocular symptoms and co-morbidities need to be taken into consideration, besides drug-related features like efficacy, route of administration and cost-efficacy. Additional considerations at the time of prescribing a treatment relate to the choice for mono-therapy versus combination treatment, nasal or oral route of drug delivery and planning of immunotherapy. Ideally, patient’s expectations, preferences and possible fear for side effects of the different treatment options should be assessed and taken into consideration in the choice of treatment in the knowledge that one treatment scheme may not be superior to the other in view of clinical efficacy. However, patients’ expectations, preferences for treatment and approach, and fear of side-effects patients are often neglected in this medical decision-making processes, in spite of the fact that these patients’ factors are an important determinant of patients' adherence and persistence to treatment ([3]) ([4]).

Evidence shows that a substantial proportion of patients with AR seem to have a clear idea about their preferred treatment for AR and visit a doctor solely to get a prescription ([5]). The role of patients’ views on disease and treatment is illustrated by a recent large-scale European survey among AR patients ([5]). This study highlighted several reasons for under-diagnosis and under-treatment: patients state that their condition is not severe enough to warrant medication, they do not feel that the allergy medication would be effective for their symptoms, they suffer from side-effects of the medication, and/or they believe that anti-allergic medications are habit-forming. These perceptions towards medical treatment may largely interfere with the control of disease, especially in regard to non-adherence and non-persistence.

At present, very little is known about patients’ perceived level of knowledge, expectations of treatment, preferences and fear for side effects for the different treatment modalities for AR. This cross-sectional explorative study therefore aims at investigating these patient-related factors in AR management, which has been a neglected area of research so far. In addition, this study also aims at exploring differences of these factors between genders and among patients with different educational levels, as the impact of these factors is unknown.

## Methods

### Design, sample and setting

This explorative cross-sectional study was conducted between July and December 2008 at the Ear, Nose and Throat Unit and Allergology Department of the University Hospitals of Leuven, Belgium. A convenience sample of 170 adult patients between 18 and 60 years of age visiting the outpatient clinic with rhinitis, without clinical evidence of rhinosinusitis after nasal endoscopy and providing written informed consent, was included in this study. Non-Dutch speaking patients were excluded, as well as patients with previous allergy testing and hence known sensitization state, as well as patients on current oral antihistamine treatment in which skin prick tests (SPT) were not performed. Patients were asked to fill in the questionnaire prior to performing the SPT, and thus were not aware of the outcome of the test when filling out the questionnaire. The study protocol was approved by the ethical committee of the University Hospitals of Leuven.

SPT were performed by trained personnel and involved the most prevalent inhalant allergens in Belgium (house dust mites, grass pollen, tree pollen and animal dander, Alternaria, Penicillium and Cladosporium, (Hal Allergy, Leiden, The Netherlands)). A SPT was defined positive by a wheal reaction of at least 3 mm in diameter or of a size equal to or larger than the positive control after 15 minutes. The diagnosis of AR was based on the ARIA criteria, involving 2 or more nasal symptoms associated with AR, and demonstration of sensitization by a positive SPT result.

### Variables and measurement

The self-report questionnaire used in this study consisted of a total of 12 items and was developed specifically for this study. The questionnaire assessed demographic and clinical variables, perceived level of knowledge, expectations of treatment, preferences in view of treatment and fear of side effects of the most common treatment options for AR. The development of the questionnaire was based on the clinical experience of the P.I. of this study (P.H.).

The following demographic variables were assessed: age (in years), gender (male/female), highest level of education (i.e. university, postgraduate eduction, high school, technical education and primary school). The clinical variable assessed was previous anti-allergic treatment (yes/no).

Perceived level of knowledge was assessed by one item. Patients were asked if they perceived themselves as having knowledge about anti-allergic treatment prior to the outpatient clinic visit (yes/no).

Expectations of anti-allergic treatment was assessed by one item. Patients were asked to indicate what their expectation of treatment was, i.e. suppression of symptoms, cure from allergic rhinitis, combination of both previous options, none of the previous options, or no opinion.

Patient preferences for treatment were evaluated by 2 items. Firstly, patients were asked to mark their preferred route of administration for their anti-allergic treatment: nasal spray, oral tablet, combination of nasal spray and oral tablet, injection treatment, none of the previous, or other treatment. Patients could only indicate one possible answer of this list. Next, the preferred treatment strategy was further explored by asking what approach they would prefer: ([1]) 'step up' approach starting with monotherapy followed by combination treated in the case of insufficient symptom control, ([2]) 'step down' approach starting with combined treatment with nasal spray and tablet for symptom relief followed by monotherapy at the time of sufficient symptom control, or ([3]) no opinion. Also here only one answer option could be indicated.

Fear of adverse events was evaluated by 4 items. Patients were asked to indicate for each of the 4 most frequently prescribed anti-allergic treatment options, i.e. nasal corticosteroid spray, oral antihistamine tablet, oral leukotriene antagonist tablet, and immunotherapy, if they feared adverse effects (yes/no/no opinion). Patients were provided with an option for free text to specify which adverse effects they feared for each of these treatments.

### Data collection and analysis

As indicated above data collection took place during a regular outpatient clinic appointment. Patients were invited by P.H. to participate in the study before the SPT was performed. After obtaining informed consent, patients filled out the questionnaires. Two investigators (V.A. and P.H.) checked completeness of the returned questionnaires.

Descriptive statistics included frequencies, means/standard deviations, medians/interquartile ranges as appropriate based on measurement level and distribution of the variable. Inferential statistics used were Fisher’s exact test. Statistical significance was set at 0.05.

## Results

### Study population

The total number of rhinitis patients included in this analysis was 170 with a balanced male/female ratio (84/86), and mean age of 40 years of age (SD: 15). SPT were performed in case of clinical suspicion of underlying sensitization. 42% (72/170) had positive SPT results, with the remaining 58% (98/170) being diagnosed as non-allergic rhinitis.

### Perceived knowledge regarding anti-allergic treatment

**Figure 1:**
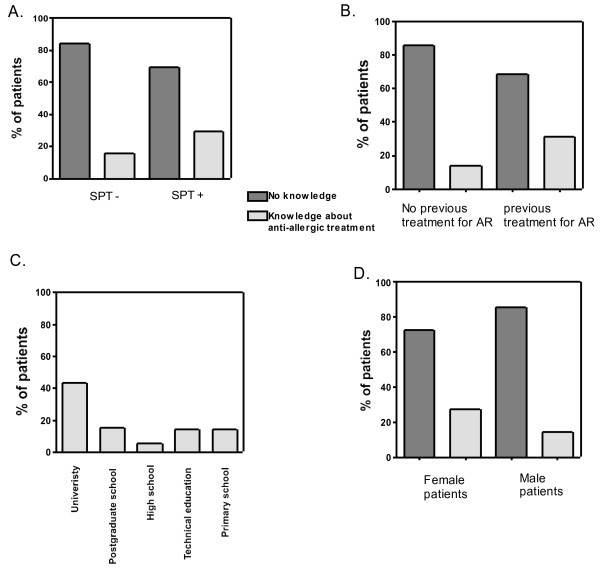
Percentages of patients reporting knowledge about allergic treatment, in relation to SPT results (negative, - vs positive, +, A), to previous treatment for allergic rhinitis (B), to highest educational level (C), and to gender (D).

Overall, 38 out of 170 rhinitis patients (22%) with suspicion of AR indicated to have knowledge about allergy treatment. A higher percentage of patients with a positive SPT (30%; 22/72) reported knowledge on allergy treatment than patients with negative SPT (15%; 16/98, Figure 1A; p = 0.04). Not unexpectedly, patients who had been treated in the past for allergic rhinitis reported more frequently (31%; 24/77, Figure 1B; p = 0.009) they had knowledge on AR treatment than patients without previous anti-allergic treatment (Figure 1B). 43% of patients with university education reported knowledge with AR treatment contrasted with the 20% of patients with less then university education reporting knowledge with ART (Figure 1C; p = 0.0006)). Female patients reported higher knowledge with AR treatment (30%; 26/86) compared to male patients (14%; 12/84, Figure 1D; p = 0.02).

### Expectations in view of anti-allergic treatment

**Figure 2:**
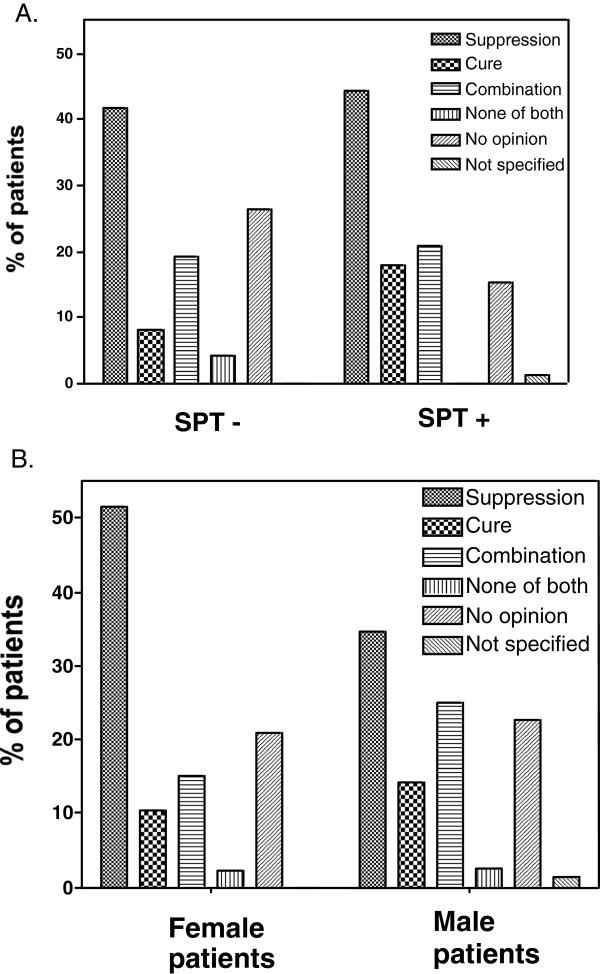
**Patients’ expectations on anti-allergic treatment, in relation to SPT results (A) and gender (B).** Data are expressed in percentages of patients expecting suppression, cure, combination of both, none of both, no opinion or unspecified.

When asked about expectations regarding allergy treatment, 43% (73/170) expected a suppression of allergy symptoms, 12% (21/170) expected to be cured from their allergy, and 20% (34/170) expected both suppression of symptoms and allergy cure. Expectations did not differ significantly between patients with and without positive SPT (Figure 2A), nor between genders (Figure 2B; p = 0.06).

### Patients' preference for treatment modality and strategy for AR

**Figure 3:**
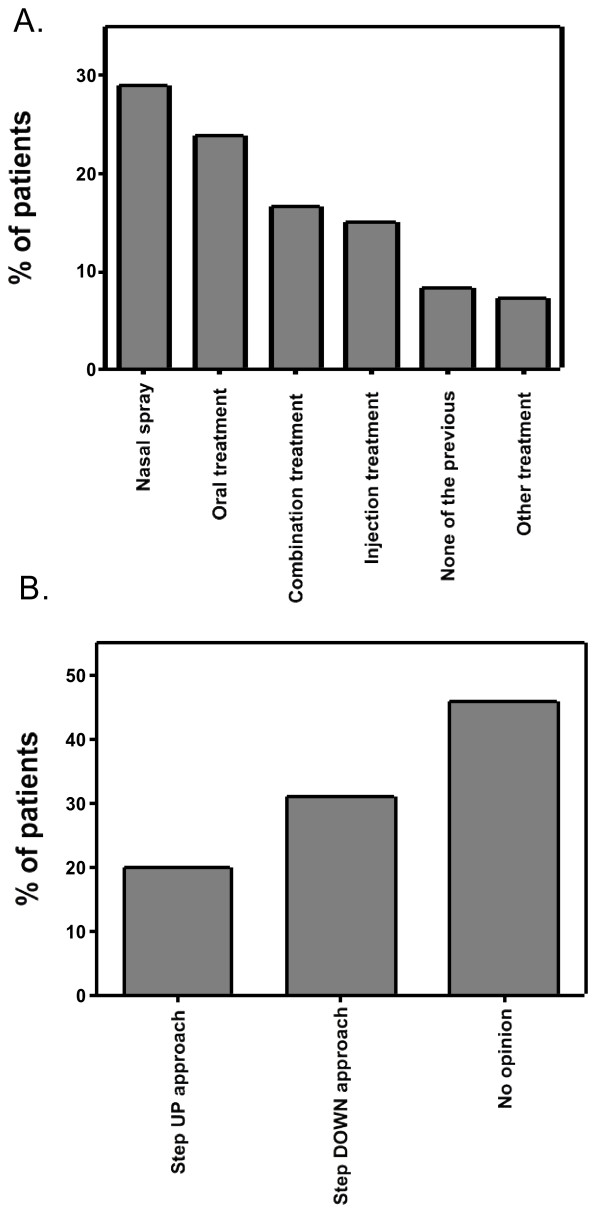
**Patients’ preferred approach for anti-allergic treatment in rhinitis.** Patients’ preference on treatment for AR: either a nasal spray, oral tablet, combination of both, injection treatment, none of previous or other treament **(A)**. Patients’ preferred strategy of treatment with either step up or step down in relation to symptom control **(B).**

Concerning the preferred route of treatment for AR (Figure 3A), 30% (52/170) preferred a nasal spray, 25% (42/170) preferred oral treatment and 16% (30/170) preferred combination treatment, whereas 15% (27/170) preferred injection treatment. The other options were rarely marked (Figure 3A).

When being asked about the preferred medical approach, 46% (78/170) of patients marked no opinion (Figure 3B). Thirty-three percent (53/170) preferred to start with a combination therapy followed by step-down, while 20% (34/170) of patients preferred the gradual step-up treatment from monotherapy until complaints are completely under control. Three percent of patients did not answer this question (5/170).

### Fear of side effects of anti-allergic therapy

**Figure 4:**
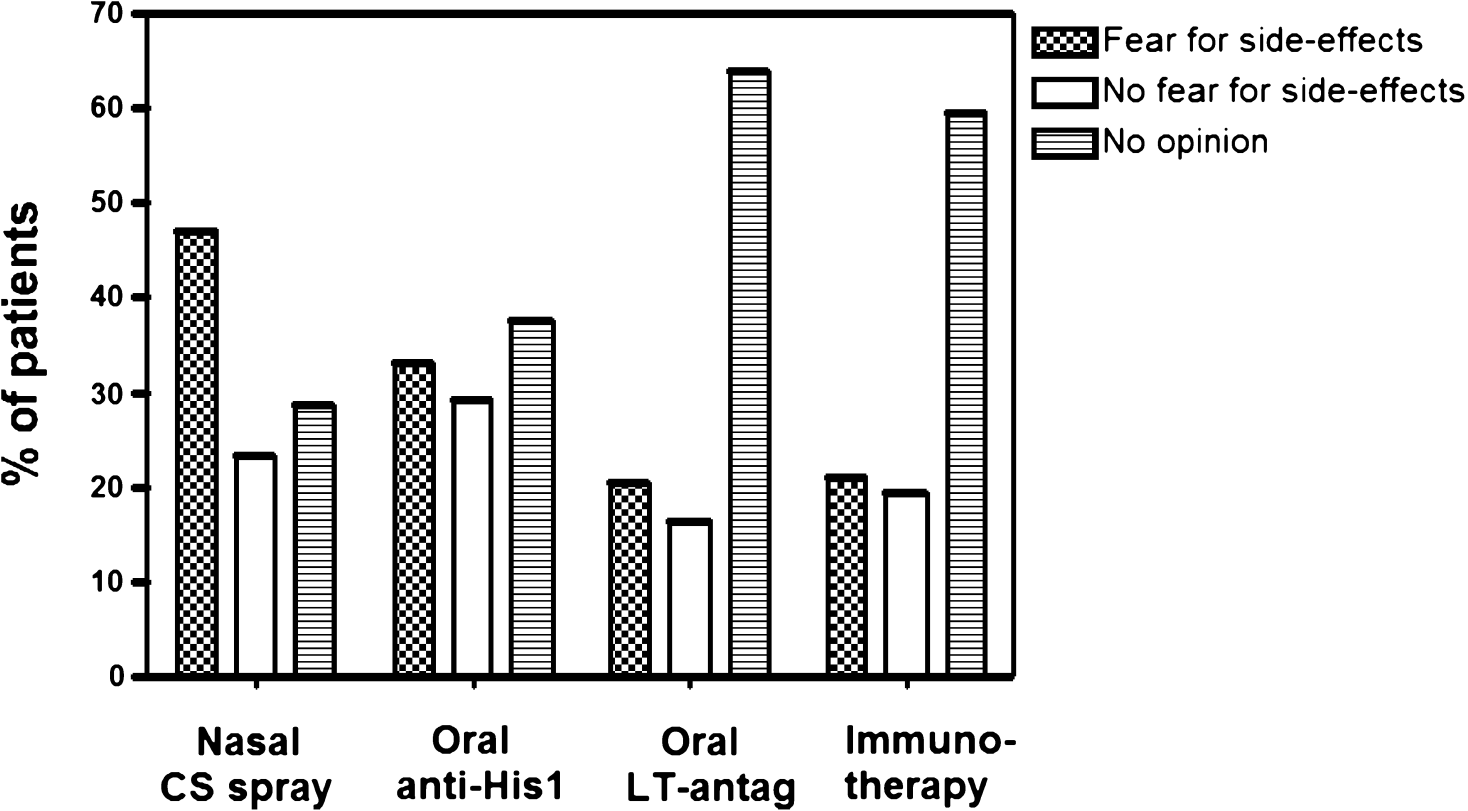
Percentage of total patients marking fear for adverse events linked to nasal corticosteroid spray, oral antihistamines, oral leukotriene antagonists and immunotherapy, no fear or no opinion.

Concerning nasal corticosteroid sprays, 48% (81/170) expressed concerns in view of side effects of this treatment (Figure 4). Thirty three percent of patients (56/170) feared side effects of oral antihistamines, 20% (36/170) did so for leukotriene antagonists, and 19% (33/170) feared side effects of immunotherapy.

Table 1 and 2 provide more detailed information what kind of specific side effects where feared by patients in the light of the nasal corticoisteroids and oral antihistamines respectively. Specific adverse events of leukotriene antagonists or immunotherapy were only mentioned by a neglectable percentage of patients (2% (3/170) and 3% (5/170) respectively) and therefore not reported.

**Table 1:** List of specific fears for adverse events of nasal corticosteroid sprays mentioned by rhinitis patients

**Fear for adverse events of *nasal corticosteroid spray* **	**(N = 24)**
Habituation	7/24 (29%)
Damage to mucous membranes	6/24 (25%)
Influence on other organs like heart, eyes, skin	6/24 (25%)
Addiction	4/24 (17%)
Weakened resistance to infection	3/24 (12%)
Weight gain	1/24 (4%)
Cancer development	1/24 (4%)

**Table 2:** List of specific fears for adverse events of oral antihistamines mentioned by rhinitis patients

**Fear for adverse events of *oral antihistamine tablets* **	**(N = 28)**
Fatigue	16/28 (57%)
Dizziness	2/28 (7%)
Habituation	2/28 (7%)
Gastric ulcer	1/28 (4%)
Interactions with other drugs	1/28 (4%)
Thirst	1/28 (4%)
Dry throat	1/28 (4%)
Dry eyes	1/28 (4%)
Fever, rash	1/28 (4%)
Addiction	1/28 (4%)
Weight increase	1/28 (4%)

## Discussion

This explorative cross-sectional study focuses on relevant patient reported outcomes in view of medical treatment for AR. More specifically, patients' perceived knowledge, expectations, preferences, and fear for side effects of AR treatment were studied. Patient’s beliefs and attitudes are important drivers in health behaviour including medication nonadherence and poor persistence in chronically ill patient populations ([6,7]). Also fear of side effects hinders patient’s engaging in prescribed treatment. So far, these factors have been underinvestigated in AR although we can infer from empirical evidence of other chronically ill patient groups that they influence patients’ motivation to start medical treatment and the impact of these factors on patients’ adherence and persistence to treatment. The latter factors are however considered to be of utmost importance in disease control ([8]).

Our findings show that 22% of patients presenting with rhinitis at ourn outpatient visit report some knowledge on anti-allergic treatment. These data strengthen therefore clinicians’ perceptions that a substantial proportion of patients already have some knowledge on the treatment of the disease. The perceived level of knowledge was highest in those who were treated previously, in patients with the highest educational level and in females. However, the correctness of this knowledge always needs to be checked in patients, irrespective of educational level or gender, as their knowledge about anti-allergic treatment may not be perfect. Indeed, the knowledge on anti-allergic treatment is often incorrect in Europe, as demonstrated by a large-scale survey. Maurer et al. studied the problem of underdiagnosis and undertreatment of patients with AR in Europe ([5]), demonstrating the presence of several false beliefs of patients on anti-allergic therapy like lack of availability of effective medical treatment and habit-forming effect of anti-allergic therapy.

It had been demonstrated before that patients with AR have a low level of satisfaction with their treatment for AR ([9]). This is not a surprise in the light of the current data showing high expectations of anti-allergic treatment. A large portion of patients expected to be cured from their allergy, or to have suppression of their complaints. Interestingly, there seem to be subtle differences in gender when it comes to expectations of anti-allergic treatment. More men expected an actual cure for their disease, whereas women tended to more frequently expect a suppression of their symptoms. The lesson to be drawn from these observations is to clearly discuss with the patient the goal of treatment of AR, albeit symptom reduction by medical treatment or alteration of the immune system and hence cure by immunotherapy. Therefore, it seems important to address unrealistic expectations in patients by explaining the underlying etiology, mechanisms and aims of anti-allergic treatment.

Concerning the way of treatment for AR, a nasal spray was preferred above oral treatment, combination treatment and injection therapy (Figure 3A). Therefore, treating the affected organ in AR seemed for most patients the preferred medical approach in AR. The anti-allergic treatment as combined treatment, with step down to monotherapy at the time of symptom control, was preferred over monotherapy with eventual combination treatment at the time of insufficient symptom control with monotherapy. This patient preference might be influenced by the severity of the symptoms, previous treatment experience, and type of medical center. Being a tertiary referral center, the majority of patients included in this trial were at the most severe spectrum of allergic disease, i.e. moderate to severe persistent allergic rhinitis, explaining in part why most patients would chose a combination treatment rather than monotherapy.

In spite of the availability of novel corticosteroids with low bioavailability, high receptor specificity and selectivity, there still exists a strong prejudice against the use of corticosteroids amongst patients ([10]). We illustrate here that 48% of our study population is concerned about adverse effects when using a nasal spray containing corticosteroids. The adverse events associated with nasal corticosteroid treatment ranged from habituation, to local damage to systemic adverse events. A similar fear for side effects was reported for oral treatment with antihistamines, but there appears to be more confidence since only 33% expressed their concern about the presence of fatigue as major side effect. Indeed, fatigue was a common adverse events in the first generation anti-histamines, but is no longer an issue in the newer second generation antihistamines. Taken together the large portion of patients fearing adverse events, medical doctors treating patients with AR should have a clear view on the potential adverse events of any of the prescribed drugs for AR and discuss with these with patients the unrealistic fear for adverse events. Concerning leukotriene receptor antagonists and immunotherapy, the patient does not seem to fear these options as much as the latter, without specification of adverse events. We estimate that the latter two treatment options are less well known amongst the general popultion, explaining the lack of fear of adverse events in most rhinitis patients.

As in every observational study, some methodological shortcomings are present. First, we only enrolled a highly selected group of patients consulting a tertiary care center for diagnosis and treatment. It remains unclear if knowledge and patient preferences would be similar in other healthcare settings, e.g. primary care. Moreover, about 42% of the study population had positive SPT results, revealing a higher percentage of allergic disease in the patients consulting the department of Otorhinolaryngology and Allergology than in the general population ([1]), relfecting a referal bias. Given the fact that the SPT results were not disclosed to patients at the time of completion of the questionnaire and knowledge levels were not different between those patients with and without positive SPT results, we decided not to omit the information obtained in the patients with non-allergic rhinitis. For the sake of clarity, we have chosen to limit the questionnaire to the most relevant questions on anti-allergic treatment involving the most commonly prescribed medical approaches.

In spite of the shortcomings, the results of the present study have some important clinical implications. The ultimate goal of any medical treatment is to fully control symptoms and improve quality of life in affected patients. Involving patients in the decision process regarding their treatment may enhance their self-care commitment and hence increase therapeutic adherence and efficiency. In AR, up to 96% of physicians state that taking the patients’ opinion into account increases therapeutic adherence ([11]) and patients' satisfaction with treatment. However, clinical reality often learns that patients are ill-informed, and may have false expectations and prejudices about anti-allergic therapy. It remains to be determined if investment in better support for patient self management and incorporating the patients’ view on disease and treatment will indeed result in better adherence, persistence of treatment and improved patient satisfaction.

## Abbreviations

AR, Allergic rhinitis; SPT, Skin prick test.

## Competing interests

All authors state they have no conflict of interest in relation to this study and the results described in the manuscript.

## Authors’ contributions

PH has initiated the study and designed the study protocol. PH, MP and JC have motivated patients to fill out the questionnaires prior to SPT. FD, KD and SD have contributed to the design of the study, the statistical analysis, the writing of the document and the revision of the final version. All authors read and approved the final manuscript.
